# Supplementary material for: Assessment of Glucose Lowering Medications’ Effectiveness for Cardiovascular Clinical Risk Management of Real-World Patients with Type 2 Diabetes: Targeted Maximum Likelihood Estimation under Model Misspecification and Missing Outcomes
Source: Int J Environ Res Public Health. 2022 Nov 11;19(22):14825. doi: 10.3390/ijerph192214825 (PMC9690556; doi:10.3390/ijerph192214825)
Supplement: Supplementary file 1 [file ijerph-19-14825-s001.zip › ijerph-1928122-supplementary.pdf]

Table S1. Results of the simulation study with different scenarios and the MAR mechanism on the outcome.

	LR	PS matching	PS covariate	IPTW	TMLE1 (CC)	TMLE2 (CC)	TMLE1 (IPW)	TMLE2 (IPW)
**SCENARIO 1: 20% MAR on Y, n = 1 000**								
OR	2.00	1.99	2.00	1.95	1.79	1.79	1.78	1.78
Bias	0.35	0.33	0.34	0.29	0.13	0.13	0.12	0.12
SE	0.56	0.58	0.55	0.51	0.36	0.35	0.35	0.35
95% NCI	87.7	91.3	88.0	90.7	94.0	92.9	94.5	92.0
**SCENARIO 2: 40% MAR on Y, n = 1 000**								
OR	2.08	2.06	2.07	2.00	1.82	1.82	1.81	1.80
Bias	0.42	0.40	0.42	0.34	0.16	0.16	0.15	0.14
SE	0.69	0.74	0.69	0.63	0.43	0.43	0.44	0.43
95% NCI	88.8	91.2	88.8	92.1	94.1	92.2	93.8	92.0
**SCENARIO 3: 20% MAR on Y, n = 5 000**								
OR	1.96	1.93	1.96	1.90	1.76	1.76	1.75	1.75
Bias	0.30	0.27	0.30	0.24	0.10	0.10	0.09	0.09
SE	0.35	0.34	0.35	0.30	0.17	0.18	0.17	0.17
95% NCI	60.2	70.8	61.2	72.5	90.4	89.1	90.4	89.3
**SCENARIO 4: 40% MAR on Y, n = 5 000**								
OR	2.03	2.00	2.03	1.96	1.80	1.80	1.79	1.79
Bias	0.37	0.34	0.38	0.30	0.14	0.14	0.13	0.13
SE	0.44	0.43	0.44	0.37	0.22	0.22	0.22	0.22
95% NCI	56.6	71.3	56.4	72.0	88.2	87.2	88.6	88.4

OR = odds ratio, SE = standard error, 95% NC = 95% nominal coverage interval, MAR = missing at random, n = sample size, LR = logistic regression, PS = propensity score, IPTW = inverse probability of treatment weighting, TMLE = targeted maximum likelihood estimator, CC = complete case, IPW = inverse probability weighting. TMLE1: LR with main terms only, LR with stepwise model selection procedures, LR with interaction terms; TMLE2: LR with main terms only, LR with stepwise procedures, LR with interaction terms, LR with interaction terms with stepwise model selection procedures, generalized additive models (GAM), random forest (RF) and recursive partitioning and regression trees (RPART).
